# Buccal Abscess Caused by Toothbrush Trauma: A Case Report of a Two-Year-Old

**DOI:** 10.7759/cureus.41055

**Published:** 2023-06-27

**Authors:** Eiji Naruo, Masaki Hayama, Tsutomu Sano, Yuka Yamamoto, Chisako Masumura

**Affiliations:** 1 Otolaryngology, Hyogo Prefectural Nishinomiya Hospital, Nishinomiya, JPN

**Keywords:** prevotella, eikenella corrodens, streptococcus anginosus, oral trauma, toothbrush, buccal abscess

## Abstract

Children often experience impalement trauma when they fall while holding objects in their mouths. While most cases heal without complications, here we report a case of buccal abscess formation after toothbrush trauma. A two-year-old boy fell while running with a toothbrush in his mouth, which punctured his right buccal mucosa. The following day, he presented to a pediatrician with a fever and buccal swelling and was treated with oral antibiotics. However, the buccal swelling did not improve, and the patient was referred to our department. Four days after the visit, the buccal swelling and fever worsened, requiring hospitalization, intravenous antibiotics, and drainage. The inflammation quickly disappeared following treatment, with no recurrence. Prophylactic antibiotic administration for oral impalement trauma is controversial. Our results suggest that prophylactic antibiotics covering both anaerobic and aerobic bacteria are necessary in cases of toothbrush-related oral trauma, where multiple bacterial infections may occur.

## Introduction

Children are more prone to head and facial trauma due to the relatively large size of their heads compared to their trunks; therefore, oral trauma is common in children. Combined oral and oropharyngeal trauma is estimated to account for approximately 1% of pediatric trauma cases [1]. Tumbles are the most common cause of oral trauma, but the requirement for medical attention increases if trauma is caused by foreign objects in the oral cavity [2]. Trauma with a foreign body in the mouth often results in impalement wounds, and most wounds heal without complications [3].

However, we encountered a case in which a buccal abscess developed one week after impalement trauma caused by a toothbrush. Although the use of prophylactic antibiotics for penetrating trauma of the oral cavity remains controversial, antibiotics were required in our case because of abscess formation. This case report focuses on toothbrush hygiene and discusses our findings.

## Case presentation

**Figure 1:**
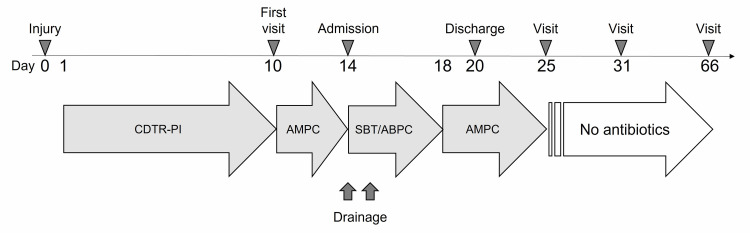
Time course of events and the antibiotics used CDTR-PI: Cefditoren pivoxil; AMPC: Ampicillin; SBT/ABPC: Sulbactam/ampicillin

The patient was a two-year-old boy with no relevant medical history. He fell while running with a toothbrush in his mouth, which got stuck in his right buccal mucosa on day 0 (Figure 1). The bleeding stopped after approximately 5 minutes, and the patient was not taken to the hospital. The next day (day 1), he experienced fever and swelling in his right cheek; thus, he was taken to his pediatrician. The oral cavity wound had almost healed, and there was a tender 2 cm x 2 cm mass on his right cheek. The pediatrician suspected hematoma and prescribed antibiotics to prevent infection. However, he was referred to our hospital on day 10 because the swelling persisted after administering cefditoren pivoxil (10 mg/kg/day, 10 days).

**Figure 2:**
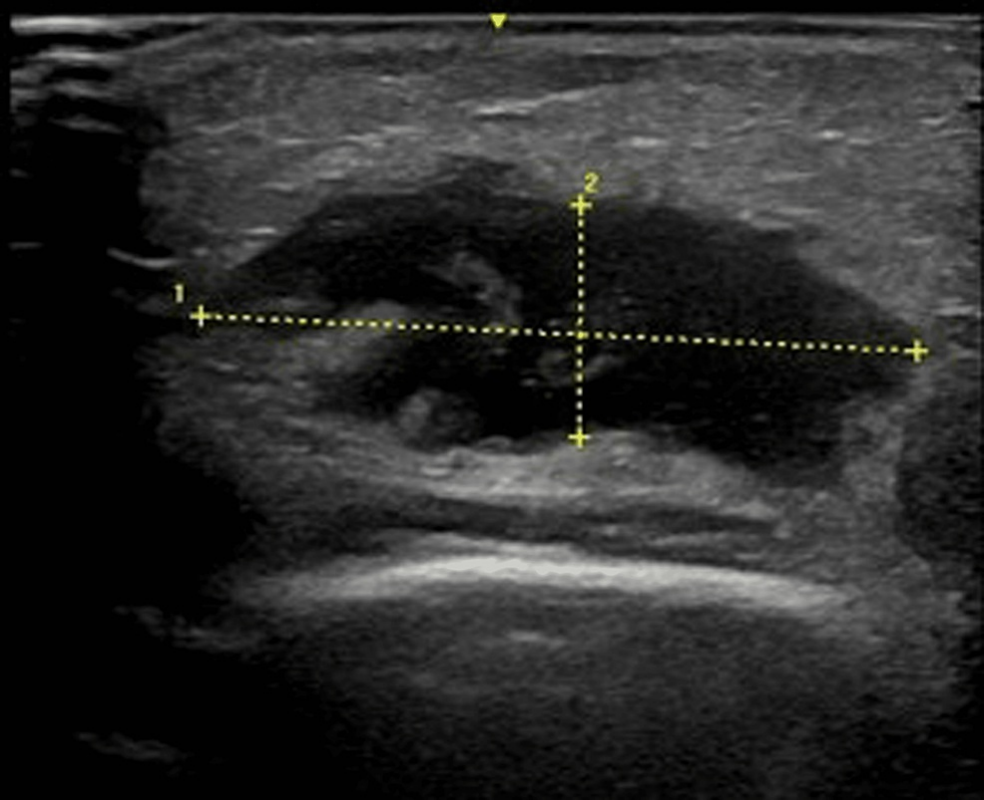
An ultrasound image of the left buccal region on the first visit A low echo area is seen (28.3 × 9.2 × 14.9 mm)

**Figure 3:**
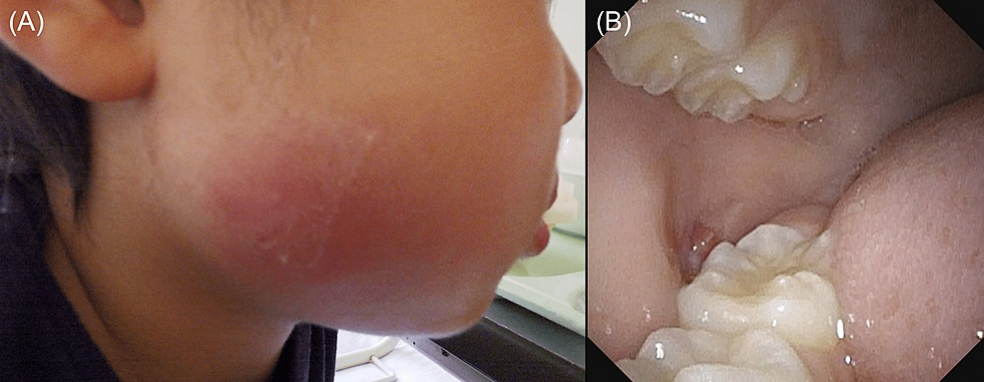
A photograph taken on the day of admission (A) The right cheek is erythematous and swollen; (B) An intraoral photograph of the right cheek

The tenderness persisted, but there was no fever, and the oral wound was not red or purulent. Ultrasound (US) examination revealed a hypoechoic area measuring 28.3 × 14.9 × 9.2 mm (Figure 2), but it was difficult to determine whether it was an abscess or an organized hematoma. The antibiotic was changed to ampicillin (20 mg/kg/day), and the patient was followed up. However, three days later (day 14), the swelling and pain increased, with associated erythema (Figure 3) and fever, requiring inpatient treatment.

**Figure 4:**
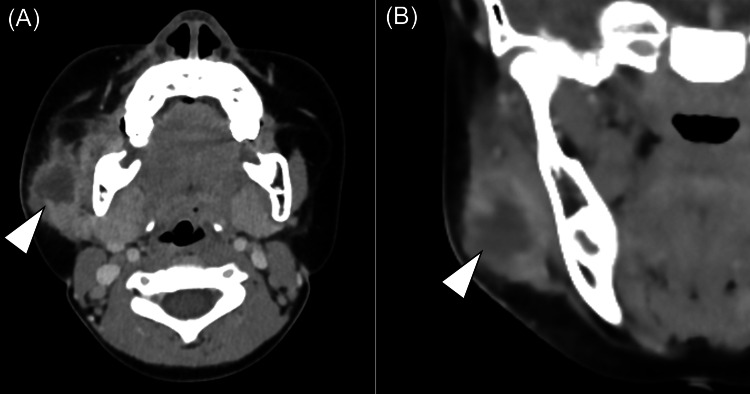
A contrast-enhanced CT scan (A) Axial image; B) Coronal image The abscess is seen in the buccal region (white arrowhead).

On the first day of admission (day 14), contrast-enhanced CT revealed a 28 × 27 × 18 mm fluid collection cavity with an irregular cap in the right cheek (Figure 4). The abscess was located lateral to the masseter muscle. In addition, increased adipose tissue density, fascial thickening, and skin thickening were observed in the submandibular region, with a slight contrast effect on the surface of the right parotid gland, suggesting spillover of inflammation. Laboratory data showed an elevated WBC count (12,100 cells/μL) and C-reactive protein level (CRP; 2.13 mg/dL). Percutaneous US-guided fine needle aspiration (FNA) was performed for a definitive diagnosis of the abscess and collection of specimens for culture, and 2 mL of pus was aspirated. The intravenous administration of sulbactam/ampicillin (100 mg/kg/day) was initiated.

On day two of admission (day 15), the US suggested a persistent abscess cavity with a diameter of 19 mm, so an FNA was performed again in the right buccal abscess, and 3.1 mL of mixed purulent and bloody fluid was aspirated. On day three of admission (day 16), the patient had no fever, and blood tests showed an improved inflammatory response with a WBC count of 6900 cells/μL and a CRP level of 0.47 mg/dL. On day four of admission (day 17), the swelling in the right cheek area improved, and the abscess was reduced to a size that did not require US-guided aspiration. On day five of admission (day 18), the right cheek swelling and oral dysfunction resolved. Subsequently, the sulbactam/ampicillin infusion was terminated, and the patient received oral ampicillin (30 mg/kg/day) for seven days. He was discharged from the hospital on day 20, with no evidence of abscess reoccurrence more than one month after discharge (day 66).

The drained pus culture was positive for *Streptococcus anginosus*, 3+; *Eikenella corrodens*, 3+; *Prevotella*/*Porphyromonas*, 3+; and gram-positive rods, 3+. Black colonies were observed on anaerobic blood agar and identified as *Prevotella* or *Porphyromonas.* However, no further identification was attempted.

## Discussion

This was a case of a buccal cavity abscess that developed after impalement trauma caused by a toothbrush. The buccal cavity is a lumen that lies deeper in the skin and platysma muscle and is shallower than the buccinator muscle. It extends from the masseter muscle posteriorly to the angle of the mouth anteriorly. This space contains various structures, such as the buccal fat pad, the parotid duct, and branches of the facial nerves. The patient presented to our department 10 days after the injury. Because of the time elapsed since the trauma, a pre-existing hematoma was initially suspected, and prophylactic antibiotics were administered. Four days later, there was an exacerbation, which was diagnosed as an abscess and treated with intravenous antibiotics and drainage. In a recent study conducted in Japan, Inoue et al. [2] reported 319 cases of oral injuries in children. In 22% of the cases, foreign objects were held in the mouth when the injury occurred, and the most common object was a toothbrush (31%). In a study of 144 children with oral impalement injuries, Matsusue et al. [3] also reported that a toothbrush was the most common culprit and that most penetrating oral traumas occur before the age of five years. The soft palate and pharynx were the most common trauma sites, and the buccal mucosa was involved in 3% to 13.2% of cases.

Furthermore, in a study by Matsusue et al. [3], none of the 131 patients with penetrating trauma developed an abscess, which suggests that abscess formation due to toothbrush trauma is rare. However, there have been reports of deep neck abscesses after penetrating toothbrush trauma [4]. Therefore, the use of prophylactic antibiotics remains controversial [5], and no guidelines exist for treating trauma to the oral cavity or pharynx.

Used toothbrushes may be colonized by various bacteria, especially when used for prolonged periods, i.e., more than 4 weeks [6,7]. Therefore, some reports recommend regular sterilization of toothbrushes [8]. In our case, several bacteria were detected in the pus culture, consistent with the results of a previous study on toothbrush hygiene. *Streptococcus anginosus*, also known as *Streptococcus milleri*, was detected in this case; it is a human commensal that colonizes the mucosal membranes of the oral cavity. *Streptococcus anginosus* may cause peritonsillar, brain, and liver abscesses [9]. *Eikenella corrodens* is a part of the oral symbionts and a normal flora of the mucosal surface of the gastrointestinal and urinary tracts [10]. Inflammation caused by *E. corrodens* is most common in the head and neck and may be associated with abscesses.

Prevotella and Porphyromonas are gram-negative anaerobes and etiologic agents of chronic adult periodontitis [11]. Porphyromonas is commonly found in the subgingival area of the teeth and has been frequently isolated from patients with periodontitis or those who have undergone root canals, as well as from healthy individuals. Exacerbating factors for abscess formation may include* S. anginosus* in case of co-colonization with *E. corrodens *or *Prevotella* [12,13] and ineffective antibiotics [12]. In the present case, oral antibiotics were initially administered prophylactically and may have been ineffective. Because a toothbrush is commonly colonized by multiple organisms, it may be more likely to exacerbate infection than other causes when involved in impalement trauma. Thus, although prophylactic antibiotic administration is controversial, prophylactic broad-spectrum antibiotic administration for anaerobic organisms may be considered in cases of impalement trauma caused by toothbrushes.

In cases of pharyngeal abscesses, including peritonsillar abscesses, a β-lactamase inhibitor-enhanced penicillin (amoxicillin/clavulanate or ampicillin/sulbactam) or a β-lactamase-resistant antibiotic combined with an anti-anaerobic drug (clindamycin or metronidazole) is recommended [14]. Since sulbactam/ampicillin was effective in our case while cefditoren pivoxil and low-dose ampicillin were not, the antibiotic recommendation for a pharyngeal abscess may also be suitable for oral abscesses.

The treatment of abscesses involves drainage and antibiotic therapy. In our case, two punctures were necessary because a single puncture was insufficient to aspirate and remove the fluid. All punctures were performed under US guidance; thus, there were no complications. Drainage using an indwelling needle cannula has been reported [14], and the method is worth trying for larger abscesses to avoid multiple punctures as it allows continuous aspiration. High-dose intravenous antibiotics may prevent the need for surgical drainage [15]. However, in this case, drainage was necessary because the abscess was large and the skin of the cheek area was likely to undergo self-destruction, resulting in cosmetic sequelae. Moreover, drainage is also important for culture and deciding which antibiotic to use. Another option would be drainage through a small skin incision. However, due to the possibility of scarring on the child’s face and the risk of facial nerve palsy, puncture drainage was considered the first choice because of its minimal invasiveness. In addition, incisional drainage often requires the placement of a drainage tube, which is difficult to manage in young children due to the possibility of accidental removal of the tube. The other drainage option is transoral incisional drainage, especially when the abscess is adjacent to an oral wound. In this case, however, the oral wound was closed and the abscess was isolated from the oral mucosa; therefore, intraoral incisional drainage was not indicated. Given that puncture drainage has been established as an alternative to incisional drainage for peritonsillar abscesses [16], which are anatomically close to the buccal cavity, it can be assumed that puncture drainage will be effective in the treatment of buccal abscesses that are not large.

## Conclusions

Although checking for vital organ damage in toothbrush trauma is important, careful attention should be paid to evidence of delayed infection, especially abscess formation. Used toothbrushes are often contaminated with multiple bacteria, including anaerobic bacteria; anaerobic bacteria co-localizing with oral commensal bacteria may exacerbate inflammation. The prophylactic administration of antibiotics covering anaerobic bacteria should be considered in cases of impalement trauma of the mouth caused by a toothbrush. Further studies, including large cohort studies, are needed to understand the clinical nature of this condition better.

## References

[1] Hennelly K (2023). Oropharyngeal Trauma in Children. https://www.uptodate.com/contents/oropharyngeal-trauma-in-children.

[2] Inoue N (2017). Oral injuries in children presenting to a Japanese pediatric emergency room. Pediatr Int.

[3] Matsusue Y, Yamamoto K, Horita S, Inagake K, Kirita T (2011). Impalement injuries of the oral cavity in children. J Oral Maxillofac Surg.

[4] Law RC, Fouque CA, Waddell A, Cusick E (1997). Lesson of the week. Penetrating intra-oral trauma in children. BMJ.

[5] Randall DA, Kang DR (2006). Current management of penetrating injuries of the soft palate. Otolaryngol Head Neck Surg.

[6] Zinn MK, Schages L, Bockmühl D (2020). The toothbrush microbiome: impact of user age, period of use and bristle material on the microbial communities of toothbrushes. Microorganisms.

[7] Raiyani CM, Arora R, Bhayya DP, Dogra S, Katageri AA, Singh V (2015). Assessment of microbial contamination on twice a day used toothbrush head after 1-month and 3 months: an in vitro study. J Nat Sci Biol Med.

[8] Naik R, Ahmed Mujib BR, Telagi N, Anil BS, Spoorthi BR (2015). Contaminated tooth brushes-potential threat to oral and general health. J Family Med Prim Care.

[9] Pilarczyk-Zurek M, Sitkiewicz I, Koziel J (2022). The clinical view on Streptococcus anginosus group — opportunistic pathogens coming out of hiding. Front Microbiol.

[10] Li L, Shi YB, Weng XB (2022). Eikenella corrodens infections in human: reports of six cases and review of literatures. J Clin Lab Anal.

[11] Wakabayashi H, Kondo I, Kobayashi T, Yamauchi K, Toida T, Iwatsuki K, Yoshie H (2010). Periodontitis, periodontopathic bacteria and lactoferrin. Biometals.

[12] Udaka T, Hiraki N, Shiomori T, Miyamoto H, Fujimura T, Inaba T, Suzuki H (2007). Eikenella corrodens in head and neck infections. J Infect.

[13] Shinzato T, Saito A (1994). A mechanism of pathogenicity of "Streptococcus milleri group" in pulmonary infection: synergy with an anaerobe. J Med Microbiol.

[14] Tanaka K, Tsunoda A, Tou M, Sonoda K, Arai S, Anzai T, Matsumoto F (2020). Minimally invasive and inexpensive percutaneous abscess drainage using an indwelling needle cannula. Am J Otolaryngol.

[15] Wong DK, Brown C, Mills N, Spielmann P, Neeff M (2012). To drain or not to drain — management of pediatric deep neck abscesses: a case-control study. Int J Pediatr Otorhinolaryngol.

[16] Menegas S, Moayedi S, Torres M (2021). Abscess management: an evidence-based review for emergency medicine clinicians. J Emerg Med.

